# A Study on Group Key Agreement in Sensor Network Environments Using Two-Dimensional Arrays

**DOI:** 10.3390/s110908227

**Published:** 2011-08-25

**Authors:** Seung-Jae Jang, Young-Gu Lee, Kwang-Hyung Lee, Tai-Hoon Kim, Moon-Seog Jun

**Affiliations:** 1 Department of Computer Science, Soongsil University, Sangdo-Dong, Dongjak-Gu, Seoul 156-743, Korea; E-Mails: ad3927@ssu.ac.kr (Y.-G.L.); mjun@ssu.ac.kr (M.-S.J.); 2 Department of Internet Information, Seoil University, Seoildaehak-gil 22, Jungnang-Gu, Seoul 131-702, Korea; E-Mail: dreamace@seoil.ac.kr (K.-H.L.); 3 Department of Multimedia, Hannam University, 133 Ojung-dong, 133 Ojeong-dong, Daedeok-gu, Daejeon 306-791, Korea; E-Mail: taihoonn@hnu.kr (T.-H.K.)

**Keywords:** sensor network, group key agreement, two-dimensional array, key pre-distribution, quorum system

## Abstract

These days, with the emergence of the concept of ubiquitous computing, sensor networks that collect, analyze and process all the information through the sensors have become of huge interest. However, sensor network technology fundamentally has wireless communication infrastructure as its foundation and thus has security weakness and limitations such as low computing capacity, power supply limitations and price. In this paper, and considering the characteristics of the sensor network environment, we propose a group key agreement method using a keyset pre-distribution of two-dimension arrays that should minimize the exposure of key and personal information. The key collision problems are resolved by utilizing a polygonal shape’s center of gravity. The method shows that calculating a polygonal shape’s center of gravity only requires a very small amount of calculations from the users. The simple calculation not only increases the group key generation efficiency, but also enhances the sense of security by protecting information between nodes.

## 1. Introduction

With the emergence of the concept of ubiquitous computing, the importance of sensor networks has become increasingly apparent. Sensor networks are bound in wireless infrastructure and recognize the situation through installed sensors and connect the recognized data to the application service [1]. Sensor nodes are becoming available for use in more fields as long distance communication becomes possible and low cost, low electricity and high capacity sensor nodes are now being mass produced [2].

There have been studies on many aspects of sensor network security so far, but in order to guarantee the basic security of wireless channels, there needs to be a different approach from that used in the existing wireless networks. Not only the common weaknesses of the existing wireless communication systems, but also the unique characteristics of sensor networks makes it hard to implement diverse security schemes. The existing encryption technology should be made lightweight and should be realized [3].

In consideration of the sensor network environment characteristics, this paper suggests a mechanism that considers security and real time traits and requires minimum amounts of calculation. This mechanism creates group keys and identity keys using a keyset pre-distribution of two-dimensional arrays and uses a preliminary random number distribution method that performs One Time Password (OTP) functions.

The remainder of this paper is composed of Section 2, which discusses the group key based key management method and quorum system needed for group key and identity key creation, and Section 3 which suggests group key creation and a group key exchange algorithm using two-dimensional arrays. Section 4 consists of the suggested system realization and system analysis and is followed by Section 5, which presents the conclusions.

## 2. Related Work

### 2.1. Key Pre-Distribution Schemes

Key pre-distribution schemes based on symmetric cryptography have been proposed as the best security framework for sensor networks. However, such schemes have been found to be vulnerable to a novel type of attack, replication attacks. A replication attack is an attack in which adversaries can insert some additional replicated hostile nodes into the network after obtaining some secret information from the captured nodes. As the result, even a single sensor node might allow an adversary to gain partial or even full control of the network by cloning the nodes. Thus, the cloned nodes are likely to have common keys or secret shares with neighboring nodes. Therefore, a replication attack introduces significant security threats to key pre-distribution schemes.

Random key pre-distribution protocols have recently been developed to address the key establishment problem in sensor networks. Eschenauer and Gligor [4] first proposed the basic random key pre-distribution for sensor network. Chan *et al.* [5] improved this scheme by introducing the concept of q-composite key pre-distribution and the random pair-wise keys schemes into sensor networks. Du *et al.* [6] proposed a new key pre-distribution scheme based on Blom’s scheme [7]. A similar method was also developed independently by Liu and Ning [8].

In this paper, considering the characteristics of the sensor network environment, a simple calculation is used through a two-dimension array key set pre-distribution. Thus, this simple calculation not only increases the group key generation efficiency, but also prevents replication attacks, which are a potential threat after node capture events in the existing random key pre-distribution schemes.

### 2.2. Group Key Based Key Management Method

Group key based key management method is a method of using group keys to maintain security within the group when geographically nearby nodes form and operate groups together [4]. The most representative method is a group key management scheme with a basic center aggregator structure, and with a base station and cluster structure at the core. Among such group key related studies, the research conducted by Deng, Han, and Mishra is particularly noteworthy [9,10]. In that study the assumption is that beforehand each sensor node has a 1:1 secret key with a base station and a three-type mechanism that would deliver safe group key with a single echo hash function and uTESLA [11–13].

**Figure 1.**
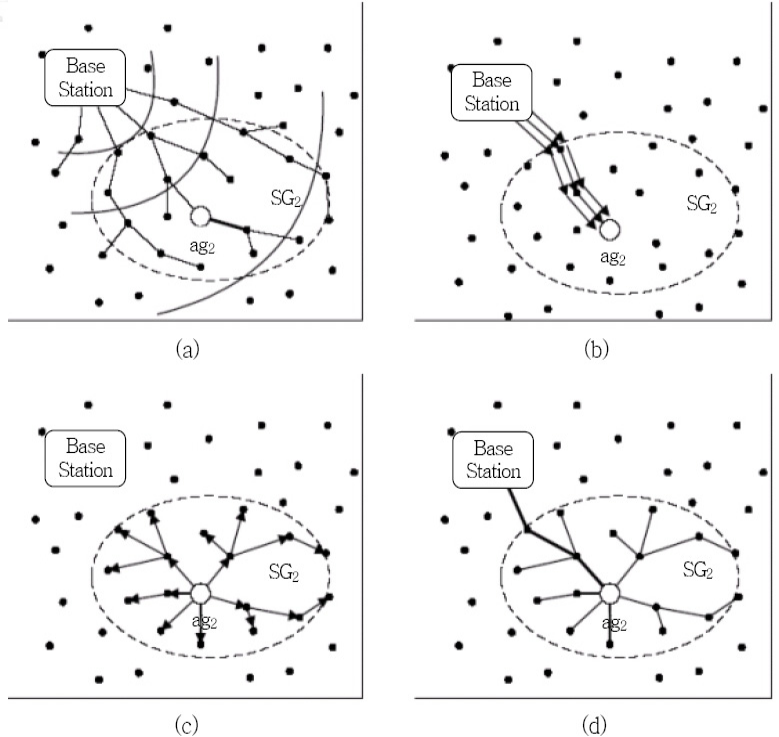
Group key management method.

Figure 1 shows each stage of this method. The first stage (a) refers to the group announcing stage where the groups are formed beforehand, an aggregator is selected and the base station broadcasts aggregator information for each group to all sensor nodes. In the second stage (b), the base station delivers nodes and group keys to the aggregator of each group. Stage (c) involves the aggregator of each group delivering a group ID and group key to nodes in the respective group. The last stage (d) is where each node recognizes the group they belong to individually and where they safely send the group key.

### 2.3. Quorum System

Quorum can be defined with more than one element of the two intersections [14]. In other words, if two intersections have been arbitrarily calculated, the meeting of gathering that does not have an empty set can be called a quorum system. For instance, {1,2,3,4,}, {2,5,6,8}, and {3,8,9,0} is a quorum system. Intersection calculation with {1,2,3,4} ∩ {2,5,6,8} = {2}, {2,5,6,8} ∩ {3,8,9,0} = {8}, or {1,2,3,4} ∩ {3,8,9,0} = {3} does not create an empty set and thus is a quorum system. If the groups are {1,2,3,4,}, {2,5,6,7}, and {3,8,9,0}, intersection of {2,5,6,7} and {3,8,9,0} would be an empty set and thus, this would not create a quorum system.

### 2.4. Grid Quorum System

**Figure 2.**
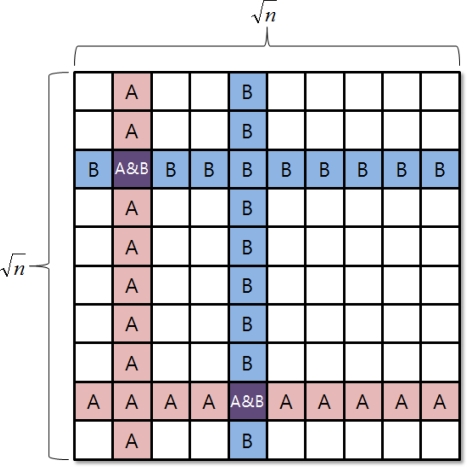
Grid Quorum System.

In the grid quorum system, the elements are aligned on the two-dimensional level field. To configure a set, one for each row and column is selected. Figure 2 shows a two-dimensional level field in the grid quorum system. The elements on the selected column and row shall be collected and be designated as the elements of one’s own. Such gathering shall follow certain rules and shall be arbitrarily designated. Arbitrarily chosen two gatherings within the system will show at least two intersections. Such a quorum system will select $2\sqrt{n}-1$ elements among the total number of elements n and will guarantee more than two common elements [15].

## 3. Group Key Agreement Using a Two-Dimensional Array

### 3.1. Term Definitions

The following terminology is used in this paper:

- BS: Base Station
- SN_A, SN_B, SN_C: Sensor Node A, Sensor Node B, Sensor Node C
- ID_A_, ID_B_, ID_C_: each sensor node’s identifier
- X_BS_ : base station’s row value         Y_BS_: base station’s column value
- X_A_, X_B_, X_C_: each sensor node’s row values  Y_A_, Y_B_, Y_C_: each sensor node’s column values
- r: random number               new_ r : new random number
- h( ): one-way hash function
- ⊕:XOR calculation
- Encryption_key( )_: symmetric key encryption algorithm

### 3.2. Group Key Agreement System

The system suggested in this paper consists of a BS and SNs. The BS takes the core role of creating and distributing group keys when forming group networks. Each SN and BS is assumed to have a random number r that has OTP role and a keyset that can create keys.

**Figure 3.**
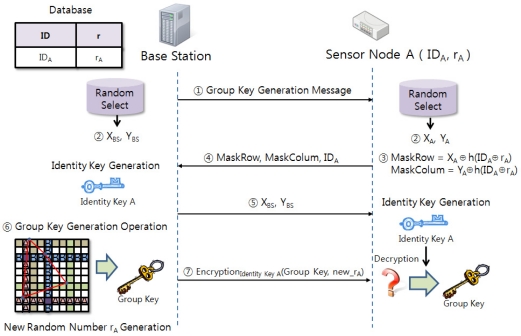
Group Key Agreement System.

Figure 3 shows the system configuration. The group key creation and distribution process are as follows:

1. BS sends a Group Key Generation Message to SN_A.
2. BS and SN_A randomly select one column and row from the keyset.
3. SN_A creates MaskRow and MaskColumn values by calculating hash and XOR with sensor node A’s identifier, random number and column and row values.
  (1) $$\begin{array}{c} \text{MaskRow}=X_{A}⊕h\left({\text{ID}}_{A}⊕r_{A}\right)\\ {\text{MaskColumn}=Y}_{A}⊕h\left({\text{ID}}_{A}⊕r_{A}\right) \end{array}$$
4. SN_A sends IDA, MaskRow and MaskColumn values to the BS.
5. BS sends column and row values to SN_A that are necessary to create Identity Key A.
6. BS creates a random number rA that is to be newly shared with the Group Key.
7. The created Group Key and new_rA will be encoded as the Identity Key A and delivered to SN_A.

**Figure 4.**
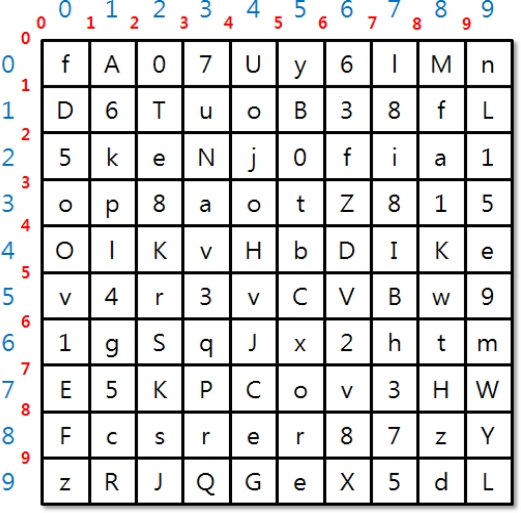
Keyset for Group Key Creation.

The BS and SNs receive a keyset in the two-dimensional array shown in Figure 4. Separately delivered keysets can input a 10 × 10 value and each cell can input capital letters A–Z, small letters a–z, and numbers 0–9. The cells will be filled with random functions.

The BS and SNs perform calculations to create group key and identity key with the keyset. The two-dimensional field has been divided into numbers that represent columns and rows and numbers represent lines that divide cells. Using such double grid will create a grid space that allows drawing shapes while being a two-dimensional array field.

### 3.3. Group Key Creation Algorithm

**Figure 5.**
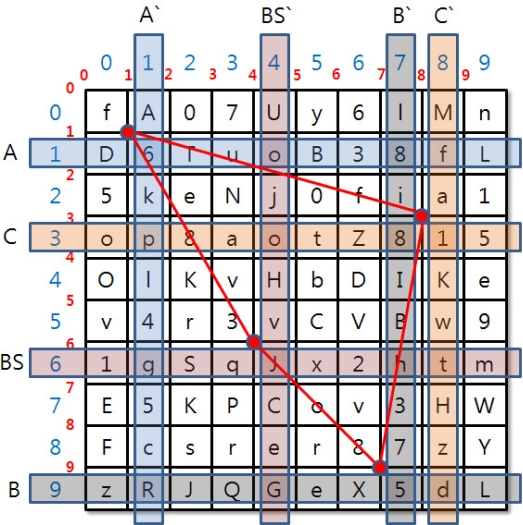
Keyset work for the group key extraction.

A group that has three SNs (A, B, C) is assumed. The BS and each SN create a group key through the following procedure:

1. BS sends a group key creation message to each SN.
2. BS and each SN randomly select a column and row from the keyset.
  (2) $$\begin{array}{c} A\prime s\;\text{selection}:\;\text{keyset}\left(X_{A},Y_{A}\right)\\ B\prime s\;\text{selection}:\;\text{keyset}\left(X_{B},Y_{B}\right)\\ C\prime s\;\text{selection}:\;\text{keyset}\left(X_{C},Y_{C}\right)\\ \text{BS}\prime \;\text{selection}:\;\text{keyset}\left(X_{\text{BS}},Y_{\text{BS}}\right) \end{array}$$
3. MaskRow, MaskColumn value and ID are sent to the BS:
  (3) $$\begin{array}{l} A\prime \text{s send}:\;\text{IDA},\;\text{MaskRow}=\text{XA}⊕h\left(\text{IDA}⊕\text{rA}\right)\;\text{MaskColumn }=\text{YA}⊕h\left(\text{IDA}⊕\text{rA}\right)\\ B\prime \text{s send}:\;\text{IDB},\;\text{MaskRow}=\text{XB}⊕h(\text{IDB}⊕\text{rB}),\;\text{MaskColumn }=\text{YB}⊕h\left(\text{IDB}⊕\text{rB}\right)\\ C\prime \text{s send}:\;\text{IDC},\;\text{MaskRow}=\text{XC}⊕h(\text{IDC}⊕\text{rC}),\;\text{MaskColumn }=\text{YC}⊕h\left(\text{IDC}⊕\text{rC}\right) \end{array}$$
4. BS confirms random number r through the ID value and deducts column and row values from the MaskRow and MaskColumn value sent by the SNs.
5. BS goes through process of deducting these values from own keyset.
  Figure 5 shows the keyset work for the group key extraction.
  The extracted values are displayed in Table 1.
6. A group key is created using a hash function by obtaining the center of gravity value of the shape drawn with the selected values and coordinates:
  (4) Group Key=Hash[6||5||1||J||{Centroid(x,y)}]
  The BS will obtain the group key after the above procedure.

### 3.4. Group Key Exchange Algorithm

**Figure 6.**
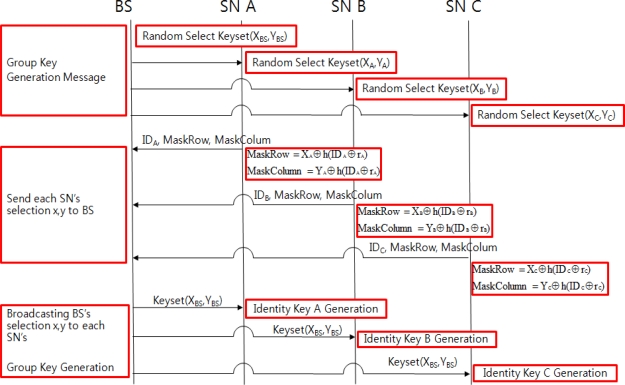
Group Key Creation and Identity Key Exchange Algorithm.

In order to safely send the group key to each SN, confidentiality, integrity and non-repudiation, *etc*. need to be guaranteed. Safe group key distribution requires encoding the created group key whereby the group member uses the same key used in encoding to conduct decoding the encoded group key. In order to achieve that, there needs to be a method to safely deliver the key used in the encoding and this is accomplished by the so-called key exchange algorithm. Figure 6 shows the order of steps required for identity key exchange.

The BS will send a group key creation message to each SN and BS and each SN will randomly select a column and row from one’s keyset. Next, the procedure of each SN sending its ID, MaskRows and MaskColumn values to BS is the same as that of the group key creation procedure. The BS with the created group key will go through the following steps after the group key exchange process to safely deliver the group key to each SN.

1. The BS that received column and row values delivered by each SN, and SN_A, SN_B, SN_C that received column and row value selected by the BS shall extract intersection elements from the keyset.

**Figure 7.**
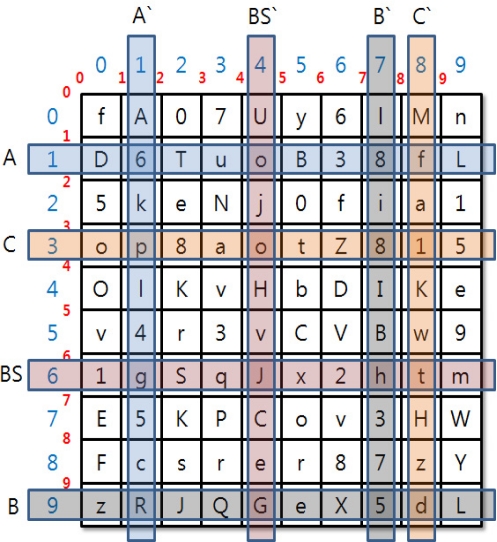
Identity Key Creation Method.

Figure 7 shows how to extract intersection elements from the keyset. For example, column and row values of BS, SN_A, SN_B and SN_C are (6, 4), (1, 1), (9, 7), (3, 8). The set of values that belongs to each column and row is a set of each group. Intersection elements are extracted from each group and the BS’ set.

The extracted values are displayed in Table 2.

(2) Identity key is created using a hash function with the calculated intersection element.
  (5) $$\begin{array}{c} \text{Identity Key A}=\text{Hash}\left(o||g\right)\\ \text{Identity Key B}\;=\;\text{Hash}\left(J||G||e\right)\\ \text{Identity Key C}\;=\;\text{Hash}\left(o||t||1||H\right) \end{array}$$
(3) The BS delivers the group key and the new random number r that were encoded by identity key to each SN.
  (6) $$\begin{array}{l} {\text{Encryption}}_{\text{Identity}\_\text{Key}\_A}\left(\text{Group}\;\text{Key},\;{\text{new}\_r}_{A}\right)\\ {\text{Encryption}}_{\text{Identity}\_\text{Key}\_B}\left(\text{Group}\;\text{Key},\;{\text{new}\_r}_{B}\right)\\ {\text{Encryption}}_{\text{Identity}\_\text{Key}\_C}\left(\text{Group}\;\text{Key},\;{\text{new}\_r}_{C}\right) \end{array}$$
Each SN that receives the encoded group key will perfom the decoding with its own identity key and will obtain a random number r that is to be newly shared with the group key, which will be used for actual data delivery.
  (7) $$\begin{array}{l} {\text{Decryption}}_{\text{Identity}\_\text{Key}\_A}\left(\text{Group}\;\text{Key},\;{\text{new}\_r}_{A}\right)\\ {\text{Decryption}}_{\text{Identity}\_\text{Key}\_B}\left(\text{Group}\;\text{Key},\;{\text{new}\_r}_{B}\right)\\ {\text{Decryption}}_{\text{Identity}\_\text{Key}\_C}\left(\text{Group}\;\text{Key},\;{\text{new}\_r}_{C}\right) \end{array}$$

## 4. System Realization and Analysis

### 4.1. System Realization

The system to be used for function evaluation, which is based on the suggested system, is realized using C# of Intel(R) Core™2 Quad CPU Q9400 @ 2.66 GHz 2.67 GHz, RAM 3.00 GB, and Window 7’s Ultimate K 32 bit operating system. The hash function needed for group key and identity key creation used the SHA-1 function, whereas the AES symmetric key encoding algorithm has been applied to encode the created key and delivery message.

**Figure 8.**
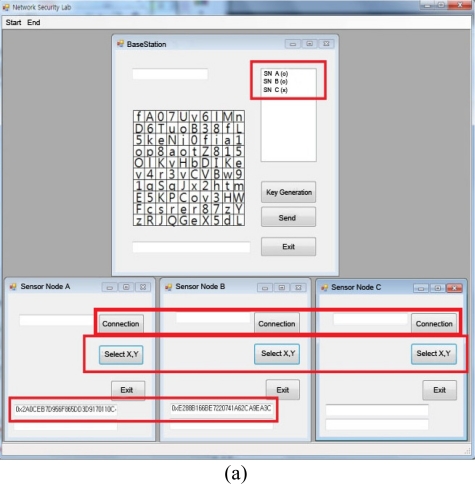

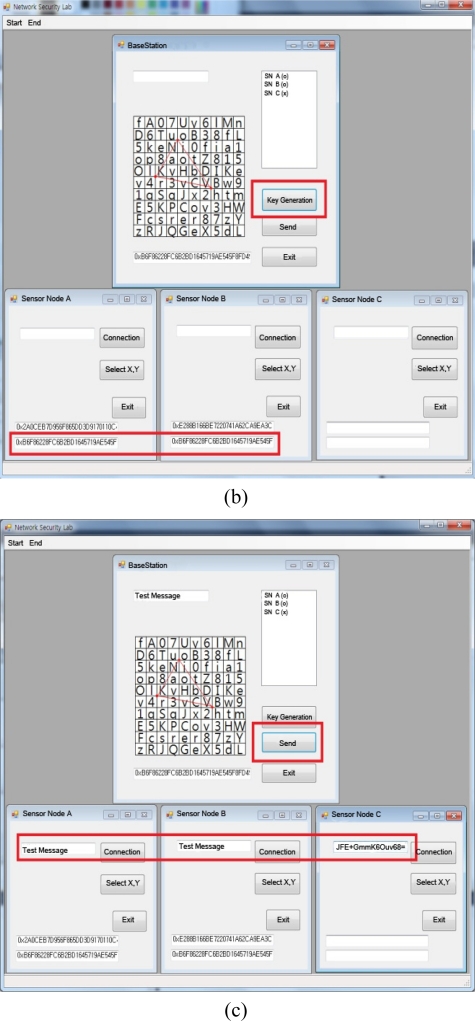
System realization.

The experiment is composed of a BS for group key creation and sensors SN_A, SN_B and SN_C that previously had a keyset and a random number r. Each SN will connect to the BS and deliver the selected values (column and row) to it, which will determine SN_A and SN_B as the close nodes and create a group key by forming a SN_A,B group. Delivering the message after encoding it with the group key to each SN_A, SN_B, and SN_C via the created group key will allow normal message display through decoding the encoded message sent to SN_A and SN_B that are connected to the group; SN_C, which is not connected to the same group, can only look up the encoded message. Figure 8 shows each stage of system realization.

Figure 8(a) displays SN_A and SN_B creating the identity key with BS determining only SN_A and SN_B as groups, even though SN_A, SN_B, SN_C are all connected and send the selected X and Y values. Figure 8(b) shows the group key delivery encoded in each SN’s identity key when the group key creation button is pressed on the BS. SN_C shall not receive the group key since it is not included in the group.

Figure 8(c) is the screen shown when “Test Message” text is sent to SN_A, SN_B, and SN_C from the BS. Here, SN_A and SN_B that have the group key will receive the original text by decoding the encoded message, but SN_C that does not have the group key will receive a non-recognizable message since it is incapable of decoding the encoded text.

### 4.2. System Analysis

The key creation method using personal information such as ID or MAC and IP is the simplest method, but it allows easy exposure or change of such information in case of a malicious user. In other words, since the user certification direction is one-way, it may be exposed to risks such as personal privacy invasion. On the other hand, the Diffie-Hellman method or the suggested system conducts a two-way certification for user certification and it can be considered safe from fraud identification attacks and is also safer than other methods with regard to personal privacy issues since it does not involve using personal information for key creation.

In general, a hash function used for key creation methods due to their quick calculation speed. This paper has also used a hash function for key creation in which a value necessary for key creation is deduced within a two-dimensional array closed field. For instance, creating a key using a hash function after selecting four values will create n! number of possible keys and will thus create 4! different keys. However, the suggested method in this paper will create not a 4! but 5! number of keys since a polygonal shape’s center of gravity value is calculated and added despite selecting only four values. In other words, selecting n number of values will create (n + 1)! possibilities, which will make it difficult for the malicious user to recognize the key. Here, polygon represents a node of sensor group and their center of gravity is used with the coordinates to create group key.

Table 3 is a comparison according to the number of vertices for the necessary time to calculate the polygonal shape’s center of gravity value needed in the group key creation. As shown in the table, a triangle shows an average value of 8.21 ms, a square 8.7 ms, a pentagon 8.87 ms, a hexagon 8.81 ms, and a heptagon with 7 points and average of 8.73 ms. What we can see here is that the amount of calculation will not increase to a great extent, despite the increasing number of the vertices and it consumes under 0.1 s to find the center of gravity for a polygonal shape.

**Figure 9.**
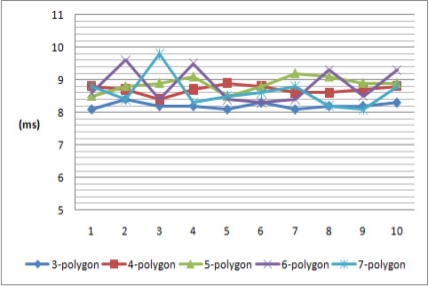
The necessary time to calculate a polygonal shape’s center of gravity.

Figure 9 is a graph version of Table 3 and makes it easy to recognize that the average speed of calculating the center of gravity of a polygonal shape is approximately 8.5 ms. As a result, the method increases the group key generation efficiency. Therefore it can be applied to various sensor environments.

## 5. Conclusions

The sensor network environment emphasizes limits on calculation capability, storage mechanisms and electronic mechanisms with the formation of wireless networks between nodes. Its purpose is to create a lighter version of the existing mechanism. Also, the characteristics of wireless communication make it vulnerable to spoofing, reply and replication attacks.

In consideration of the characteristics of the sensor network environment, this paper creates a group key through a keyset pre-distribution of a two-dimensional array which will satisfy the demands for confidentiality, certification, and integrity between BS and SNs. The proposed mechanism will also increase efficiency and security by using a preliminary random number distribution method that conducts OTP role.

In the proposed scheme, the two-dimensional array consists of a double coordinate. BS and SN get the necessary values from a two-dimensional array to create a group key. This should minimize the exposure of keys and personal information. The key collision problems are resolved by utilizing a polygonal shape’s center of gravity. The method that uses a double coordinate array and shape information regarding the center of gravity shows that calculating a polygonal shape’s center of gravity requires a very small amount of calculation for the users. It shows the possibilities for future adaptability to any type of environment that requires various values that do not overlap within the confined resources and where the domain is not limited to group key creation.

## Figures and Tables

**Table 1.** Extracted Value to KeySet.

**Group’s Value**	**Column**	**Row**	**SEED VALUE**
A’s Value	1	1	6
B’s Value	9	7	5
C’s Value	3	8	1
BS’ Value	6	4	J

**Table 2.** Intersection Element.

**Group**	**Set**	**∩ BS**
A	{D, 6, T, u, o, B, 3, 8, f, L, A, k, p, I, 4, g, 5, c, R}	{o, g}
B	{z, R, J, Q, G, e, X, 5, d, L, I, 8, i, B, h, 3, 7}	{J, G, e}
C	{o, p, 8, a, t, Z, 1, 5, M, f, K, w, H, z, d}	{o, t, 1, H}
BS	{1, g, S, q, J, x, 2, h, t, m, U, o, j, H, v, C, e, G}	

**Table 3.** The necessary time to calculate a polygonal shape’s center of gravity.

	**3-polygon**	**4-polygon**	**5-polygon**	**6-polygon**	**7-polygon**
1	8.1ms	8.8 ms	8.5 ms	8.6 ms	8.8 ms
2	8.4 ms	8.7 ms	8.8 ms	9.6 ms	8.4 ms
3	8.2 ms	8.4 ms	8.9 ms	8.4 ms	9.8 ms
4	8.2 ms	8.7 ms	9.1 ms	9.5 ms	8.3 ms
5	8.1 ms	8.9 ms	8.5 ms	8.4 ms	8.5 ms
6	8.3 ms	8.8 ms	8.8 ms	8.3 ms	8.6 ms
7	8.1 ms	8.6 ms	9.2 ms	8.4 ms	8.8 ms
8	8.2 ms	8.6 ms	9.1 ms	9.3 ms	8.2 ms
9	8.2 ms	8.7 ms	8.9 ms	8.5 ms	9.1 ms
10	8.3 ms	8.8 ms	8.9 ms	9.3 ms	8.8 ms
Average	8.21 ms	8.7 ms	8.87 ms	8.81 ms	8.73 ms
